# Digital representation of youth' agency in culture: a database of projects

**DOI:** 10.3389/fpsyg.2025.1716164

**Published:** 2025-12-12

**Authors:** Pavel S. Sorokin, Veronika D. Novikova, Mikhail E. Goshin

**Affiliations:** Laboratory for Human Capital and Education Research, Institute of Education, HSE University, Moscow, Russia

**Keywords:** youth agency, culture, cultural projects, digital traces, database, agency effects

## Introduction

Agency is a multidisciplinary concept denoting individual ability to initiate and carry out actions that transform one's own life and social environment (Bandura, 2001; Emirbayer and Mische, 1998), drawing on foundations in social cognitive (Bandura, 1986), social identity (Tajfel and Turner, 1986), and empowerment theories (Zimmerman, 2000). Traditionally, in psychology, agency is viewed as an internal characteristic—a constellation of motives, attitudes, and cognitive schemas that drive human action (Bandura, 2001). Studies of this sort focus on the desire to be active, the aspiration for autonomy, self-esteem, while often overlooking real, objective activity that exerts influence on social structures (Cavazzoni et al., 2021; Sorokin and Redko, 2024). “Strong” forms of agency, when individuals or groups directly create or transform social structures remain less studied—both within and beyond psychological literature (Hogget, 2001; de Haan and Rotmans, 2018). Yet it is precisely these forms of agency that become critical in times of increasing social tensions and institutional turbulence making individual initiative necessary for sustaining collective wellbeing (Conceicao, 2024). The forms of such agentic behavior span from corporate, technological or social entrepreneurship and volunteering to various project activities, including those performed by youth.

Digital technologies have created entirely new space for the formation and manifestation of agency (Sorokin, 2025). Social networks, platforms and online communities offer new opportunities for self-expression that might have a transformative impact on social reality (Frade, 2025; Siddiq et al., 2024). The concept of “hybrid agency” (Livingstone and Sefton-Green, 2016; Sorokin, 2023, 2025) reflects individuals' potential to combine personal initiative with the capabilities of digital platforms. For instance, hosting a podcast and building a follower community in the digital sphere using Artificial Intelligence tools (like chat-bots) enables individual self-expression and also facilitates new social connections and possible joint activities.

In this context, the sphere of culture is of particular importance. Unlike other domains of agency (e.g., technological entrepreneurship or political activism), culture is more accessible to young people, requiring neither substantial initial financial investments nor high risk (Sorokin et al., 2025a). Examples of youth projects in culture manifesting agency include preserving cultural heritage, supporting communities and creating new products, services, markets, and landscapes (see Beel et al., 2017; Amit and Wulff, 1995). Importantly, “external effects” of projects on the social environment (mentioned above) and “processual dimension” of agency (stable system of interrelations, regular events) should be distinguished (Lund and Vestøl, 2020).

Digital traces, including publications, comments, photos, and videos disseminated through media, including social networks, represent an important yet understudied source of data that can be used to document structural aspects of human agency—in particular, in such legitimate and easily accessible field as culture (Frade, 2025; Siddiq et al., 2024).

Despite the growing scholarly interest in youth' agency (Cavazzoni et al., 2021; Conceicao, 2024), including in the field of culture, still lacking is a publicly available database documenting the digital representation of youth' cultural projects at the national level, including its structural forms and effects. This Data Report presents a database of youth-led cultural projects, incorporating 94 initiatives across Russia. The dataset contains specially structured information on these projects. Examples include restoring and preserving cultural heritage, shaping and supporting cultural identities, and transforming public spaces—in line with existing literature on single cases representing other locations (for instance, for Albania—see Menkshi et al., 2021).

In contrast to other studies on youth agency, including cultural projects, this database was constructed using a novel methodological approach based on analyzing digital traces of youth-led cultural projects. This dataset may be of particular interest to scholars of cultural and social psychology, as well as those studying transformations in the post-Soviet space.

## Methods

### Data collection period, sources and search strategy

The dataset was collected between January 2025 and June 2025 in the major digital platforms for representation and support of youth' initiatives in Russia, including Social Media VKontakte, messenger Telegram, social media Instagram and specialized web-sites aimed at discussing and supporting youth projects in culture. The data collection process was conducted intwo stages.

In the first stage, relying on existing experience in research on human agency representation in the digital environment in Russia (Sorokin et al., 2025a; Sorokin et al., 2025b), using both automatic and manual tools we screened more than 50 projects relating to culture, identified in major publics in Social Media VKontakte. We searched for the cultural projects using keywords: “culture,” “fashion and style,” “craft,” “entertainment,” “cultural centre,” “artist,” “theater,” “entrepreneur,” “gallery,” “public association,” “creative spaces,” “street art,” “art residencies,” “art clusters,” etc.

We revealed different types of cultural projects, which were grouped into three broad categories: (1) other-oriented cultural entrepreneurship (projects aimed at providing opportunities for others to perform novel cultural practices and produce cultural content), (2) self-oriented cultural entrepreneurship (producing self-authored creative products or services changing cultural landscape), and (3) cultural volunteering (supporting cultural initiatives). Each category demonstrates a different pattern of agency representation, which resulted in partially different inclusion criteria (according to each parameter, selected based on previous research) (Sorokin et al., 2025c).

The second stage consisted of sample formation (using the same mentioned above keywords) and verification using various sources, including VKontakte, Telegram, and Instagram. Only projects with systematic representation of their activities over the past 9 months (in terms of the digital traces they generate) were included. Every potentially relevant project that we identified was searched for additional digital representation in other sources of digital data using the projects' name as a keyword (e.g., Telegram, Instagram, and specialized web-sites).

### Inclusion criteria

Projects were included in the database according to the following criteria:

1. The frequency of novel cultural content production in VKontakte/Telegram/Instagram: at least 10 times per month for other-oriented cultural entrepreneurship; five times per month for self-oriented cultural entrepreneurship; three times per month for cultural volunteering.
2. Audience engagement on the digital platform (VKontakte/Telegram/Instagram):

- For other-oriented cultural entrepreneurship: minimum 2,000 subscribers, average of at least 15 likes per post, and at least three comments per post;
- For self-oriented cultural entrepreneurship: minimum 600 subscribers, at least eight likes per post;
- For cultural volunteering: minimum 900 subscribers, average of at least 10 likes per post, and at least two comments per post.

3. Explicit identification of the project leader, core team members, partners, or affiliated initiatives in public digital sources.
4. Affiliation with cultural themes, including contemporary art, local cultural heritage, etc.
5. Project leader aged 18–35 at the time of data collection.
6. Minimum project duration of 3 years, verified through consistent digital content publication and activity traceable across at least 36 months.

Projects *were not considered* if they were purely commercial with no explicit declaration of social orientation or mission, or if they were directly affiliated with and fully controlled (as can be visible from their digital traces) by the state or corporate entities.

These criteria are justified by the authors' 5 years long (since 2021) experience in several large scale empirical research projects (both applied and fundamental) focusing on agency representation and manifestation in the digital field. During these projects more than 3,000 projects were analyzed while authors worked closely with entrepreneurs, volunteers, and project-leaders. This experience is reflected in a number of peer-reviewed articles in leading journals (see, for example, Sorokin, 2025).

### Data description

The database includes 94 projects from 37 regions of Russia. Each project was described according to the following key variables (see Table 1). Project self-descriptions (digital profiles, websites) were studied and compared with actual manifestations, representing not only declarations from the project team but also the impact on the social environment visible through digital traces: social media activity, audience engagement (comments, reposts, discussions), and the information disseminated by media and other digital communities. Interpretation was carried out independently by three researchers, followed by consensus-building based on analytical notes. Records were initially composed in Russian, then translated into English by bilingual researchers, with key fragments verified through back-translation to ensure accuracy.

**Table 1:** Structure of dataset variables.

**Variable**	**Description**
Project name	Name
Geographic region	Region/s where the project demonstrates activity
Description	Brief description
Target audience	The scale of major target audience (local/regional/Russian residents/Foreigners)
Forms of digital representation	VK group, Telegram, YouTube, personal blog, etc.
Declared agency effects (detailed description)	The projects' mission and goals as stated on the projects' website and/or profile in Social Media
Observed agency effects (detailed description)	Actual (empirically observable through digital traces) effects from media, reports, reviews
Structural effects of the project (typological)	-*Stimulating collective action aimed at common good*-*Educational effects: skill-building for initiative and mobility*-*Empowering at-risk groups to overcome discriminations*-*Supporting from below transformations initiated by the dominant institutions*-*Introducing innovative cultural products or practices by connecting the past with present*

## Youth agency in culture: extended opportunities and created identities

As a first step in exploration of the Data Base, we tried to outline, on the one hand, the most common features of all the selected projects (through the prism of their effects) and, on the other hand, several types of specific effects, distinguishing different projects from one another. The process involved each of the three co-authors independently reading the whole Data Base and preparing suggestions (analytical notes) from the “Description,” “Declared Agency Effects,” and “Observed Agency Effects” variables, based on personal interpretation. Following discussion and comparison, the authors iteratively grouped the suggestions into overarching themes and specific agency effects, ultimately reaching a consensus through negotiation.

The first common feature of the selected projects is their *use of the digital sphere as an extended field of agency*. Here, youth demonstrate initiative by constructing a cultural space around themselves. They run channels, write reviews, organize online meetings and workshops, manage communities, and build digital identities. Each post or broadcast is an act of self-expression and an invitation to engage in dialogue, inspiring and eliciting a response. Thus, the Internet becomes a space for expanded agency: young people are not just users, but also content creators, moderators, and initiators.

The second common feature uniting the majority of the projects is *the search for and construction of the “self” using cultural narratives and tools*. Many projects (“Favna,” “Antifragility,” “Lok Tatchö,” and “Dom Kustarya Cultural Center”) are aimed at restoring ethnic, regional, or cultural identity. By participating in field research, processing oral histories, reconstructing rituals, and engaging in dialogue with local communities, young people come into direct contact with heritage—not as museum artifacts, but as living, dynamic parts of their own biography. In addition to these two general effects, there are several more specific effects identified in particular groups of projects.

1. *Stimulating collective action aimed at common good*. Projects such as “Sopki 21A,” “Guslitsa Creative Estate,” and “Stankozavod” are collective initiatives in which agency is manifested through the group action rather than individually. Together, these projects revitalize abandoned spaces, create art installations, organize festivals, and engage in dialogue with local communities.
2. *Educational effects: skill-building for initiative and mobility*. Projects such as “Residence “Art Tram,” which aims at cultivating public speaking skills, and “Fasadvik,” which teaches staff to engage visitors in dialogue with exhibits, function as spaces where agentic behavior is cultivated by developing particular skills, which support the capacity to perceive oneself as the subject of action endowed with initiative, responsibility, and the power to influence the surrounding world.
3. *Empowering at-risk groups to overcome discriminations*. These projects support individuals who are facing structural constraints, such as gender stereotypes, mental health conditions, social isolation, or the trauma of exclusion, to regain control over their lives. Through creative practices (embroidery, drawing, sculpture, and writing), participants not only master a craft, but also assert their presence as social actors by making the products and the creative process visible. For instance, the projects “Embroidery as Resistance to Stereotypes” and “Outsiderville” are intended to combat marginalization.
4. *Supporting from below transformations initiated by the dominant institutions*. Many projects, such as “Vspomnit Vse” receive some institutional support from the state, or from the non-commercial and private sector. The critical question is whether a project can preserve its internal motivation and capacity for self-determination given an external influence. As far as their digital profiles demonstrate, all the selected projects in the dataset retain the initial grassroots agentic impulse and use the external support as a tool for achieving their initial goals rather than allowing sponsors to guide their activity.
5. *Introducing innovative cultural products or practices by connecting the past with present*. These are entrepreneurial projects offering innovative cultural products and practices that bridge the identities and narratives from the past with the present socio-cultural reality—thus, producing significant structural effects (“Rodina,” “Favna,” and “Alice Muse”). The leaders of such projects explicitly declare innovative orientations, which not only meet the demands of existing markets but also create new market niches by offering novel products or services, inspired by the cultural heritage of a particular territory or region. Promoting local traditions, the founders of such projects simultaneously preserve and reinvent them, offering products or practices that resonate with the present tastes and demands.

### Data curation

The dataset was edited to ensure consistency, reliability, and reusability. The following steps were performed:

- Verification: each entry was reviewed independently by at least two co-authors, and any discrepancies in interpretation were discussed and resolved by consensus.
- Standardization: descriptions were edited for stylistic uniformity and length. Manifestations of agency were categorized according to a typology presented above.
- Anonymization: no personal data were collected; only publicly available information was used.
- Translation: all entries are provided in both Russian and English to ensure international accessibility.
- Format: data is stored and distributed via Google Sheets, and export options to CSV and Excel formats are available upon request.

## Limitations

First, all projects are extensively represented in a digital environment. Less digitally visible but potentially significant initiatives, particularly those in rural areas or closed communities, are excluded. Second, most of the data relies on self-reports from participants and organizers, which inevitably introduces an element of subjectivity. Third, the linguistic dimension also warrants attention: translating projects' information may cause the loss of nuances, especially in cases tied to ethnic, regional, or local contexts.

## Conclusion and possible research paths

This Data Report aims to objectively document youth-led cultural projects by analyzing their digital footprints. Unlike traditional case reviews, this database uses an innovative methodology based on the digital traces. The dataset helps to overcome a significant limitation of agency-related research in contemporary psychology, i.e., the tendency to analyze agency primarily as a subjective experience without focusing on its external structural forms and effects. Digital footprints are especially valuable in regard to behavioral manifestations, serving as a rich and dynamic source of evidence. The database facilitates longitudinal and comparative studies of youth agency dynamics and comparative analyses across regions, formats and social groups, all of which are grounded in over 90 “real” and “truly agentic” cultural projects.

## Data Availability

The original contributions presented in the study are included in the article/supplementary material, further inquiries can be directed to the corresponding author.
